# *In Vivo* Profiling Reveals a Competent Heat Shock Response in Adult Neurons: Implications for Neurodegenerative Disorders

**DOI:** 10.1371/journal.pone.0131985

**Published:** 2015-07-02

**Authors:** Alisia Carnemolla, Hayley Lazell, Saliha Moussaoui, Gillian P. Bates

**Affiliations:** 1 Dept. Medical and Molecular Genetics, King’s College London, 8th Floor Tower Wing, Guy’s Hospital, Great Maze Pond, London, SE1 9RT, United Kingdom; 2 Novartis Institute for Biomedical Science, Neuroscience Discovery, Basel, CH-4002, Switzerland; Emory University, UNITED STATES

## Abstract

The heat shock response (HSR) is the main pathway used by cells to counteract proteotoxicity. The inability of differentiated neurons to induce an HSR has been documented in primary neuronal cultures and has been proposed to play a critical role in ageing and neurodegeneration. However, this accepted dogma has not been demonstrated *in vivo*. We used BAC transgenic mice generated by the Gene Expression Nervous System Atlas project to investigate the capacity of striatal medium sized spiny neurons to induce an HSR as compared to that of astrocytes and oligodendrocytes. We found that all cell populations were competent to induce an HSR upon HSP90 inhibition. We also show the presence and relative abundance of heat shock-related genes and proteins in these striatal cell populations. The identification of a competent HSR in adult neurons supports the development of therapeutics that target the HSR pathway as treatments for neurodegenerative disorders.

## Introduction

The function and plasticity of specific cells relies on a distinctive proteome which is constantly challenged by intrinsic and environmental stresses. Hyperthermia represents one such stress, and heat-related pathologies, such as heat stroke, have been estimated to become one of the most serious causes of human mortality [1]. During heat stroke, a core body temperature of over 40°C causes acute tissue injury coupled with inflammatory and coagulation responses [2], leading to multi-organ failure which is often fatal [3]. This is commonly associated with permanent neurological damage in heat stroke survivors suggesting that the nervous system is particularly vulnerable [4]. Protein homeostasis (proteostasis) is critical for maintaining organismal viability and cell function. Protection against proteotoxicity involves four main stress response pathways: the heat shock response (HSR) [5], the unfolded protein response in the endoplasmic reticulum [6], the unfolded protein response in the mitochondria [7] and the oxidative stress response [8]. These stress pathways together with the ubiquitin proteasome system and autophagy represent the proteostasis network [9]. The inability of differentiated neurons to induce a heat shock response after hyperthermia, at the transcriptional level, has been documented in mouse and rat primary neuronal cultures [10–12]. In particular, cultured rat hippocampal neurons were characterized by the absence of heat shock factor-1 (HSF1) [10,11], whereas cultured mouse motor neurons showed an impaired ability to activate HSF1 [12]. However, an *in vivo* study of the HSR in different cell populations of the brain has not been performed.

We have recently published an extensive and in-depth analysis of the HSR in the mouse [13] showing that the brain is able to maintain an efficient HSR throughout a mouse lifespan. This suggests that either the HSR is not compromised in adult neurons or that non-neuronal cell types are responsible for the seemingly static nature of the HSR during ageing. To distinguish these possibilities, we have now isolated neuronal and non-neuronal cells from the striata of mice in which the HSR had been chemically induced through HSP90 inhibition [13,14]. We have taken advantage of the BAC transgenic mice generated through the Gene Expression Nervous System Atlas (GENSAT) project, for which extensive characterization by the GENSAT project and others is available [15–17]. We have adopted an effective FACS protocol [18] to purify mature genetically labelled striatal projection neurons (GENSAT lines *Drd1a*-EGFP, *Drd2*-EGFP), astrocytes (*Aldh1l1*-EGFP) and oligodendrocytes (*Cmtm5*-EGFP), referred to herein as Drd1a, Drd2, Astro and Oligo, respectively. Using this technique, we demonstrate the ability of all four cell populations to induce HSR in adult mice upon treatment with the brain penetrant HSP90 inhibitor, HSP990 [13,14]. We also show the presence and relative abundance of HSR-related genes and proteins in different cell populations of the striatum. The identification of a competent HSR in neurons as well as glial populations supports the development of therapeutics that target the HSR pathway as treatments for neurodegenerative disorders.

## Results

### Differentiated neurons induce HSP genes upon HSP990 treatment

There is considerable evidence to suggest that the HSR is blunted in fully differentiated neurons as a consequence of the lack of either HSF1 expression [10,11] or HSF1 activation [12]. To test this *in vivo*, we studied the HSR in adult medium sized spiny neurons (MSNs) of the striatum, as well as astrocytes and oligodendrocytes, using HSP990, an HSP90 inhibitor [13,14], to determine the extent to which these different cell populations were able to mount a canonical HSR.

We treated 4 GENSAT BAC transgenic lines (Drd1a, Drd2, Astro and Oligo) and wild-type mice at 6 weeks of age (43 ± 1 days) with a single acute oral dose of HSP990 (12 mg/kg) and tissues were harvested two hours later. At this time post-dosing, we expect to detect the induction of HSP mRNAs, but it is most likely too early to see increases in heat shock protein levels [13]. Each experiment included a total of 12–14 mice that were genotype and gender matched. In order to obtain the required number of GFP positive (GFP^+^) cells for mRNA analysis several experiments had to be performed for each line (S1 Table). Before pooling cells from different experiments, we used RT-qPCR on hippocampal mRNA to ensure that the level of heat shock protein induction was comparable between experiments and lines (S1A Fig).

**Fig 1:**
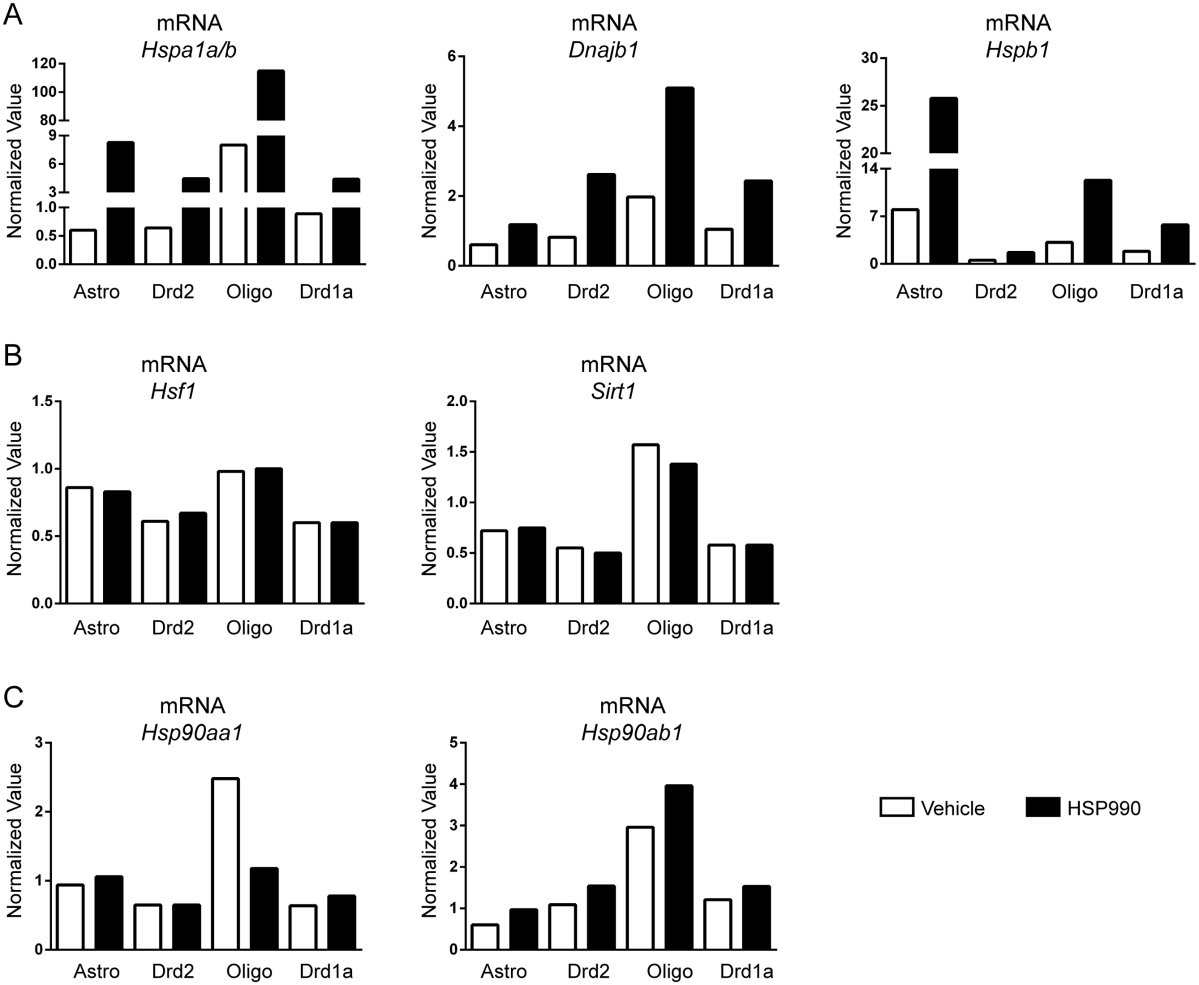
mRNA expression level of HS related genes in different striatal cell populations. Striatal cells were isolated from wild type and transgenic mice at 6 weeks of age 2 hours after treatment with HSP990 (12 mg/kg) or vehicle and sorted based on *Gfp* expression. RT-qPCR analysis of the expression levels of (A) *Hspa1a/b*, *Dnajb1* and *Hspb1* (B) *Hsf1* and *Sirt1* and (C) *Hsp90aa1* and *Hsp90ab1* in GFP^+^ cells isolated from mice treated with HSP990 as compared to those treated with vehicle.

Interestingly, all cell populations showed a clear induction of the three major heat shock protein genes, *Hspa1a/b* (HSP70) (7–15 fold), *Dnajb1* (HSP40) (~ 2.5 fold) and *Hspb1* (HSP25) (~ 3.5 fold), as assessed by RT-qPCR, and the fold induction was generally comparable between lines (Fig 1A). The identification of two housekeeping genes (*Atp5b* and *Gapdh*) that were stable across the cell populations allowed us, for the first time, to estimate the relative basal level of the HS-related genes using the vehicle treated samples. Interestingly, the levels of *Hspa1a/b* and *Dnaj1* were higher in oligodendrocytes than astrocytes and neurons, in which they were comparable, whereas *Hspb1* was more abundant in astrocytes (Fig 1A). In keeping with previous data showing that the induction of the HSR does not elicit an up-regulation of HSF1 [13,19], the expression of *Hsf1* mRNA was not modulated by HSP990 in any of the cell populations (Fig 1B). Similarly, we found no effect of HSP990 on the expression of *Sirt1* (Fig 1B), a known positive regulator of HSF1 [20], consistent with our data from whole striatum [13]. The analysis of the two HSP90 isoforms, *Hsp90aa1* (inducible) and *Hsp90ab1* (constitutive), also matched the data obtained from whole striatum [13], with no induction for *Hsp90aa1* and a mild (~1.5 fold) up-regulation for *Hsp90ab1* that did not differ between neuronal and non-neuronal cells (Fig 1C). Once more, the basal levels of *Sirt1* and the two *Hsp90* isoforms were higher in oligodendrocytes.

**Fig 2:**
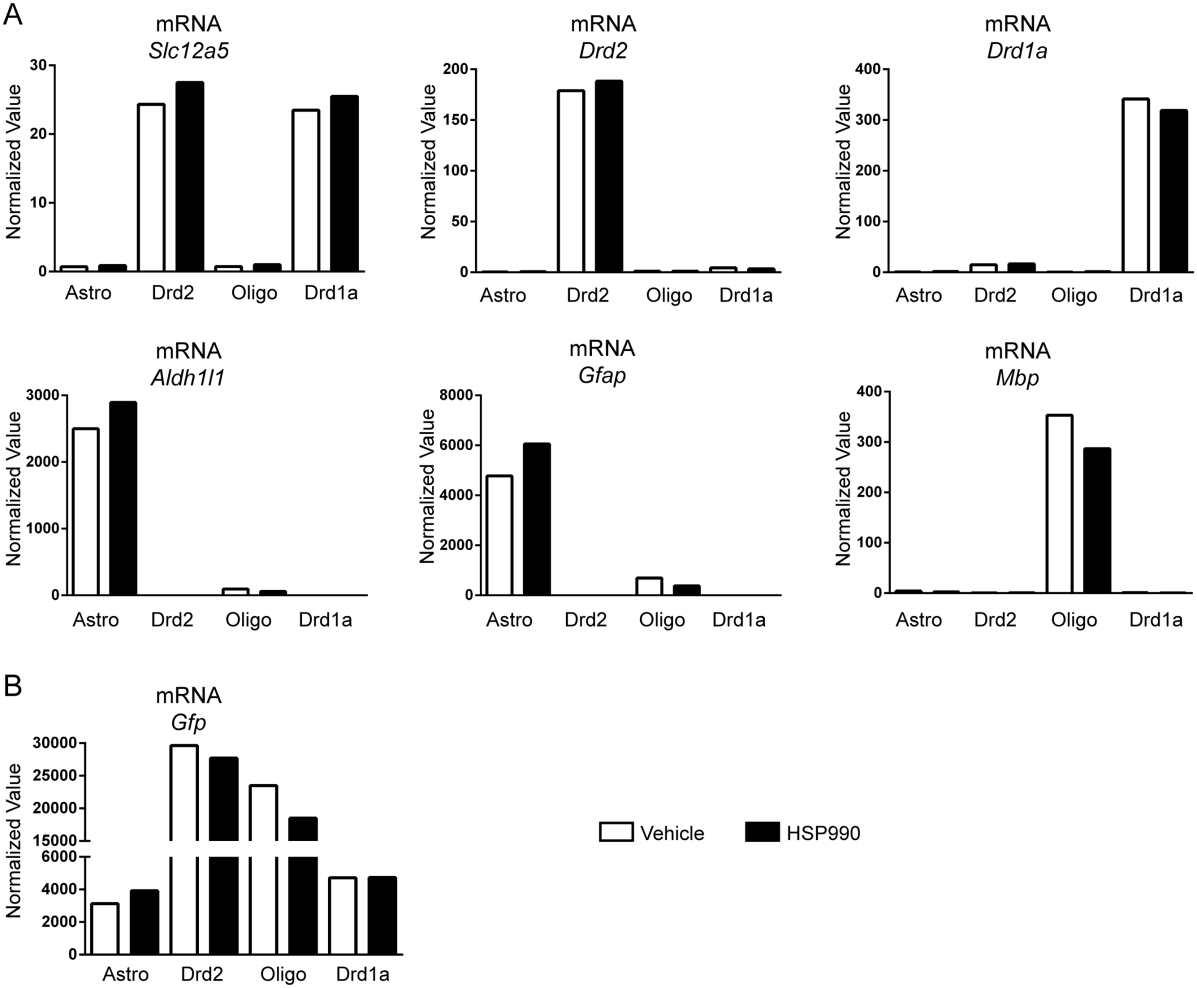
Validation of the purity of the cell populations used for the mRNA analysis Striatal cells were isolated from wild type and transgenic mice at 6 weeks of age 2 hours after treatment with HSP990 (12 mg/kg) or vehicle and sorted based on *Gfp* expression. RT-qPCR analysis of the expression levels of (A) *Slc12a5*, *Drd1a*, *Drd2*, *Aldh1l1*, *Gfap* and *Mbp* and (B) *Gfp* in GFP^+^ cells isolated from mice treated with HSP990 or vehicle.

In order to ensure cell purity, RT-qPCR was performed with markers specific for astrocytes (*Aldh1l1* and *Gfap*), oligodendrocytes (*Mbp*), neurons (*Slc12a5*) and MSNs of the direct (*Drd1a*) and indirect (*Drd2*) pathways (Fig 2). Neurons sorted from the *Drd1a* and *Drd2* GENSAT lines showed no astrocyte or oligodendrocyte contamination and were specific for the expression of their respective dopamine receptor gene (Fig 2A). The cells sorted from the Astro line were free from neuronal or oligodendrocyte contamination. Surprisingly, those sorted from the Oligo line may have shown some astrocyte contamination based on the expression of *Gfap*. However, the fact that *Aldh1I1* astrocyte marker was only detected at trace levels, may indicate that there was a low level of *Gfap* expression in the oligodendrocyte pool (Fig 2A). *Gfp* was expressed in all cell populations at levels reflecting the strength of the corresponding promoters (Fig 2B).

Taken together, these data show that fully differentiated MSNs of both the direct and indirect pathway of the striatum can trigger the induction of HSP gene expression upon HSP990 treatment. Furthermore, we were not able to detect any difference at the transcriptional level between the various cell populations of the striatum that could suggest a reduced performance of the HSR in adult neuronal cells as compared to non-neuronal cells.

### Comparative expression analysis of heat shock proteins in neuronal and non-neuronal striatal cells

**Fig 3:**
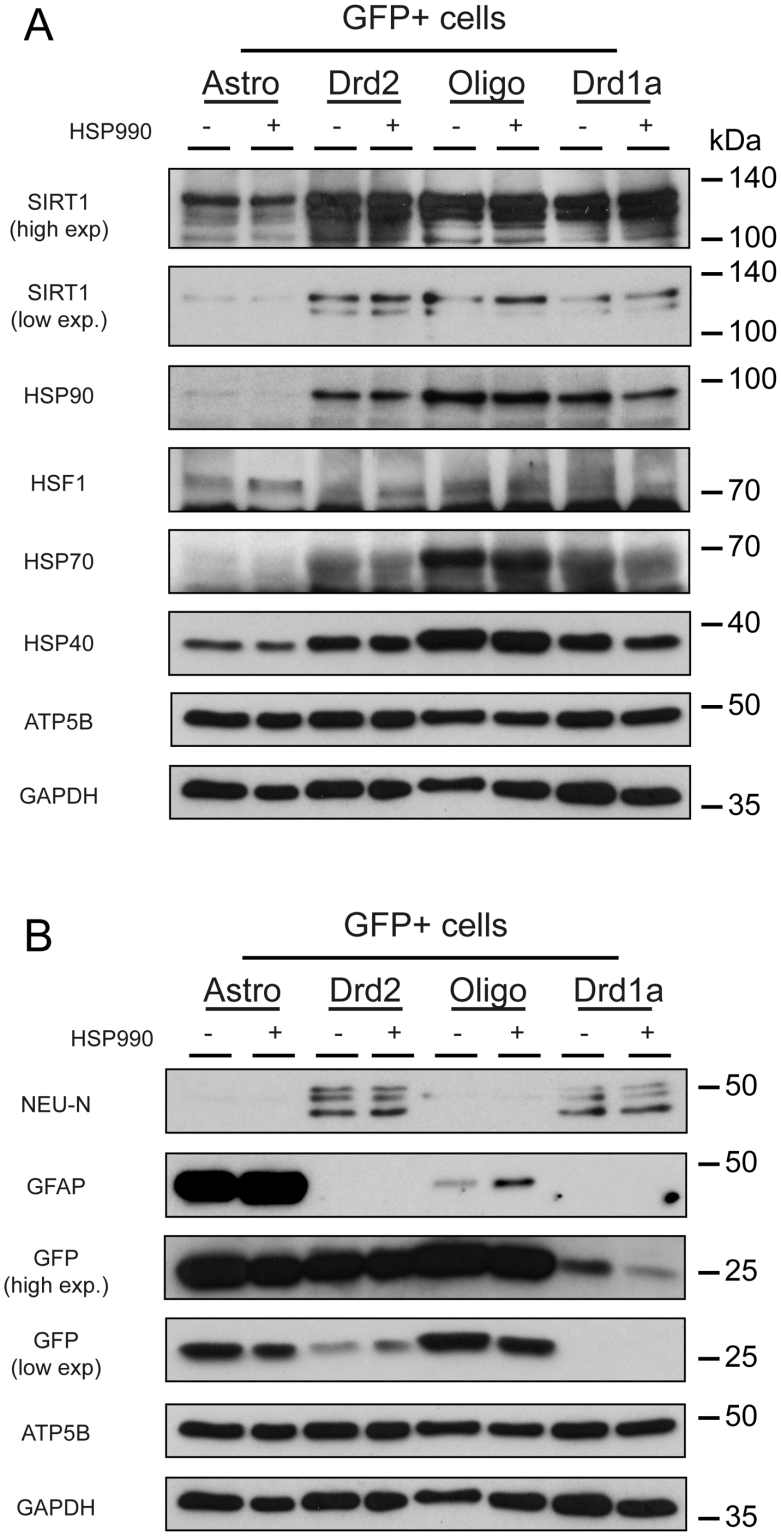
Expression of the heat shock proteins and their regulators in the striatal cell populations Striatal cells were isolated from wild type and transgenic mice at 6 weeks of age 2 hours after treatment with HSP990 (12 mg/kg) or vehicle and sorted based on GFP expression. Western blot analysis of the expression levels of (A) SIRT1, HSP90, HSF1, HSP70 and HSP40 and (B) NEUN, GFAP and GFP in GFP^+^ cells isolated from mice treated with HSP990 as compared to those treated with vehicle. Loading controls were ATP5B and GAPDH.

The next challenge was to compare the levels of the heat shock proteins in the striatal cell populations. Preliminary experiments indicated that to detect the heat shock proteins on a western blot approximately 500,000 cells would need to be collected for each cell type. Cells were harvested for sorting at 2 hours post HSP990 dosing and the number of experiments performed as well as the total number of cells collected are summarised in S1 Table. Our knowledge of the kinetics of the HSR from previous studies indicated that we would be unlikely to see induction of heat shock proteins at this time point, but we did expect to be able to compare basal levels. The collection of cells at a later time-point post-dosing was impractical due to the 5 hours required for the preparation and sorting on the cells from freshly collected tissue. As a control, before pooling cells from different experiments, we performed RT-qPCR on mRNA isolated from the hippocampus to ensure that the level of induction of HSP genes was comparable between mice (S1B Fig). In practice we were able to collect sufficient cells to detect HSP70 and HSP40, but not HSP25. As expected, we did not observe an upregulation of these chaperones (Fig 3A). Consistent with the mRNA data, the basal levels of HSP70, HSP40 and HSP90 were higher in oligodendrocytes (Fig 3A). Interestingly, SIRT1 and HSF1 were expressed in all cell populations and the levels of HSF1 mirrored the mRNA data. The HSF1 hypershift was not apparent, most likely because HSF1 had returned to its inactive state during the 5 hour preparation and sorting period (Fig 3A). As a control, to ensure that the dissociation and sorting protocol had not negatively affected heat shock protein induction, we performed western blot on protein extracted from snap frozen hippocampal tissue from each of the mice used for these experiments (S2 Fig). In keeping with the data obtained from GFP^+^ sorted cells, induction of the heat shock proteins in the hippocampal tissue was not evident at the 2 hour time point, but a clear HSF1 hypershift was observed indicative of an active transcription factor [19]. Immunoprobing with NEUN and GFAP confirmed the purity of the astrocyte and neuronal populations, whist the oligodendrocytes may either express GFAP or be contaminated with a low level of GFAP, consistent with the mRNA data (Fig 3B). All cell populations expressed GFP as expected (Fig 3B).

Taken together, these data suggest that the dynamics of translation of the HSR effectors follows the same kinetics in neuronal and non-neuronal cells of the striatum. Furthermore, this protein analysis confirms that the highest basal content of heat shock-related proteins occurs in oligodendrocytes, as was highlighted at the mRNA level. Surprisingly, the heat shock protein levels were considerably lower in astrocytes than either of the two neuronal populations.

## Discussion

The inability of mature neurons to mount a HSR [10–12] has been proposed as a critical mediator of the cell and tissue deterioration that is characteristic of neurodegenerative disorders [21]. However, whether the HSR is blunted in neurons *in vivo* had not been investigated. In the present study, we provide a detailed analysis of the HSR in multiple cell populations of the adult mouse striatum. In contrast to previous data from primary neuronal cultures [10–12], we were unable to detect an impairment of the HSR in striatal MSNs and could detect no difference in the extent to which the HSR was induced between neuronal and non-neuronal cells. Previous reports suggest that the failure to upregulate *Hspa1a/b* in hippocampal neurons was the result of the absence of HSF1 [10,11]. However, we found that in MSNs, HSF1 is expressed both at the mRNA and protein levels, although we cannot rule out that an absence of HSF1 may be specific of a hippocampal neuronal population. Overall, our data do not support a neuronal-specific blunting of the HSR in adult mice.

Our study also showed that there was a marked difference in the relative cell-specific abundance of HS-related genes and proteins: oligodendrocytes showed the highest content of chaperones and HSR regulators, whereas these levels were considerably lower in astrocytes than in either of the two neuronal populations. Intriguingly, SIRT1 was also enriched in oligodendrocytes, whereas its levels were lowest in astrocytes. Despite the controversy on the role of SIRT1 in lifespan extension, its actions on substrates known to play key roles in cellular processes related to organismal health, suggest that SIRT1 might be a central player in the biology of cells and organisms [22,23].

We took advantage of the BAC transgenic mice generated through the GENSAT project [15] to provide a means by which striatal MSNs, astrocytes and oligodendrocytes could be sorted after HSR induction by the chemical inhibition of HSP90 with HSP990. Although an effective approach, this required the pooling of several experiments to generate sufficient cells, even for the RNA analysis. More recently genetically modified mouse lines that would provide a more efficient means of isolating cell-specific mRNAs have been described [24,25]. However, isolation of sufficient cells to perform a protein analysis still remains a major challenge.

To the best of our knowledge, this analysis of the HSR in different cell populations of the striatum provides the first comprehensive picture of this cellular process in mammals. The inability of the brain to deal with chronic proteotoxic stress in neurodegenerative conditions, such as Alzheimer’s disease, Parkinson’s disease and Huntington’s disease, led to speculation that the HSR is compromised in adult neurons. Instead, we have shown that in mice, differentiated neurons are able to maintain an efficient HSR. Therefore, the development of new intervention strategies to improve the proteostasis capacity of the brain would be predicted to counteract the accumulation of misfolded proteins with beneficial consequences for neurodegenerative disorders.

## Materials and Methods

### Ethics Statement

All experimental procedures performed on mice were approved by the King's College London Ethical Review Process Committee and carried out under a Home Office License. Animals were sacrificed by cervical dislocation.

### Mouse models and maintenance

Swiss Webster mice were purchased from Harlan Olac, Bicester, UK (Hsd:ND4). The GENSAT lines [15] were obtained from the Mutant Mouse Regional Resource Centers (MMRRC) as follows: Tg(Drd1a-EGFP X60Gsat/Mmmh (MMRRC stock #297) from the University of Missouri, Drd2-EGFP S118Gsat/Mmnc MMRRC stock #230 from the University of North Carolina, and Tg(Cmtm5-EGFP) DL108 Gsat/MmucdMMRRC stock #10768 and Tg(Aldh1l1-EGFP) OFC789Gsat/Mmucd (MMRRC stock #11015) from the University of California Davis. Wild type and transgenic mice used in this study were littermates obtained by backcrossing to Swiss Webster mice. All animals were subject to a 12-hour light/12-hour dark cycle and had unlimited access to water and food. Housing conditions and environmental enrichment were as previously described [26]. Mouse striata were dissected and used fresh for each experiment; other brain regions, used for control experiments, were snap frozen in liquid nitrogen and stored at -80°C.

### NVP-HSP990 dosing

NVP-HSP990 [27,28] was obtained from Novartis Pharma AG and formulated with 0.2% methyl cellulose (Sigma, 274429) by brief sonication at high frequency and thorough mixing to form a uniform suspension. Compound or vehicle alone was freshly prepared for each round of treatment and administered to mice by oral gavage, with thorough mixing between dosing to ensure an even suspension. For all experiments, each treatment group contained age- and sex-matched mice and mice were weighed and dosed on the same day in the morning.

### Buffers and reagents for dissociation

HABGT: Hibernate-A (Brainbits, HA-pr) with 2% (vol/vol) B27 supplement (Invitrogen, 0080085SA), 0.25% Glutamax (Invitrogen, 35050038) and 5% Trehalose (Sigma, T-9531). Papain buffer: Hibernate-A minus Ca2+ (Brainbits, HA-Ca) with 0.25% Glutamax. Papain and DNase (Worthington product codes PAP2 and D2, respectively) were distributed as lyophilized aliquots.

### Dissociation of striatal cell populations into single-cell suspensions

Cell dissociation was performed as previously described in detail [18] with the exception that HABGT was used instead of HABG. A single-cell suspension was achieved by trituration with a silanized, polished glass pipette. The suspension was then filtered and centrifuged through a BSA cushion for further purification.

### Flow cytometry

Cells were sorted according to their GFP content using a FACSAriaII (BD Bioscience) with an 85 μm nozzle, at a frequency of 47.0 kHz. The 488 laser line was used for excitation. The FACSDiva “4-way Purity” purity mode was used during sorting. Dead cells were gated out using high propidium iodide (Sigma, P-4864) staining and forward light scattered. Cell yield was about 10,000–30,000 GFP positive (GFP^+^) cells per mouse for the neuronal lines, 80,000–100,000 GFP^+^ cells per mouse for the astrocyte line and 40,000–70,000 GFP^+^ cells per mouse for the oligodendrocyte line.

### Taqman RT-qPCR

RNA extraction, cDNA synthesis, Taqman RT-qPCR and ΔCt analysis were performed as previously described [29]. The Taqman qPCR assays for *Gapdh*, *Atp5b*, *Rpl13a*, *Hsp90aa1* and *Hsp90ab1* were purchased from Primer Design. For a list of primers and probes for other assays see Carnemolla et al 2014 [13].

### SDS-PAGE and immunoblotting

Frozen mouse brain was homogenized in ice cold buffer (50 mM Tris-HCl pH 8.0, 150 mM NaCl, 10% Glycerol, 1% Triton X-100, 10 mM ethylenediaminetetraacetic acid (EDTA) supplemented with complete protease inhibitors (Roche, 11697498001) and phosphatase inhibitors (1 mM sodium orthovanadate [New England Biolabs, P0758S], 50 mM NaF [Sigma, 201154], 10 nM okadaic acid [Sigma, 08010]). Frozen cell pellets were resuspended in 5% sodium dodecyl sulphate (SDS) supplemented with protease and phosphatase inhibitors. Protein concentration was determined by the BCA assay (Thermo Scientific, 23223 and 23224), and 10–20 μg protein for hippocampal tissue, or 4–8 μg protein for the cell lysates was added to 2x Laemmli loading buffer before being subjected to SDS-polyacrylamide gel electrophoresis (PAGE) and western blotting as described previously [30]. Membranes were incubated with primary antibody overnight at 4°C in phosphate buffer saline with 0.2% Tween (PBST) and 5% non-fat milk. Blots were washed 3 times for 5 min in PBST, incubated with secondary antibodies in PBST for 1 hour at RT, washed 3 times for 5 min in PBST, and exposed to ECL according to the manufacturer’s recommendations (Amersham). Signal was developed using Amersham hyperfilm and a Xenograph developer. For full details on primary and secondary antibodies see Carnemolla et al. 2014 [13]. ATP5B and GAPDH antibodies were used as loading controls.

## Supporting Information

S1 Checklist NC3Rs ARRIVE Guidelines checklist (PDF)

S1 Fig HSP gene induction in the hippocampi of mice used for the cell sorting experiments. Hippocampal tissue was isolated from wild type and transgenic mice at 6 weeks of age 2 hours after treatment with HSP990 (12 mg/kg) or vehicle. RT-qPCR analysis of the expression levels of *Hspa1a/b*, *Dnajb1* and *Hspb1* in (A) tissue collected from mice used for the mRNA analysis (Fig 1) and (B) tissue collected from mice used for the protein analysis (Fig 3). Values were calculated relative to vehicle treated wild type mice. WT = wild type, Tg = transgenic. Error bars are SEM.
(TIF)

S2 Fig HS-related protein expression in the hippocampi of mice used for the cell sorting experiments. Hippocampal tissue was isolated from wild type and transgenic mice at 6 weeks of age 2 hours after treatment with HSP990 (12 mg/kg) or vehicle. Western blot analysis of the expression levels of SIRT1, HSF1, HSP90, HSP70, HSP40, HSP25 and GFP in mice treated with HSP990 as compared to those treated with vehicle. It was possible to use GFP to confirm the genotypes of the mice used for the Astro and Oligo lines, but GFP was not expressed in the hippocampus of the neuronal lines and so could not be used for this purpose. Loading control = ATP5B. WT = wild type, Tg = transgenic. *non-specific band.
(TIF)

S1 Table The total number of mice used to generate the number of GFP+ sorted cells that were pooled for the RNA and for the protein experiments. (DOCX)
