# Supplementary figures and images for: In Vivo Profiling Reveals a Competent Heat Shock Response in Adult Neurons: Implications for Neurodegenerative Disorders
Source: PLoS One. 2015 Jul 2;10(7):e0131985. doi: 10.1371/journal.pone.0131985 (PMC4489736; doi:10.1371/journal.pone.0131985)

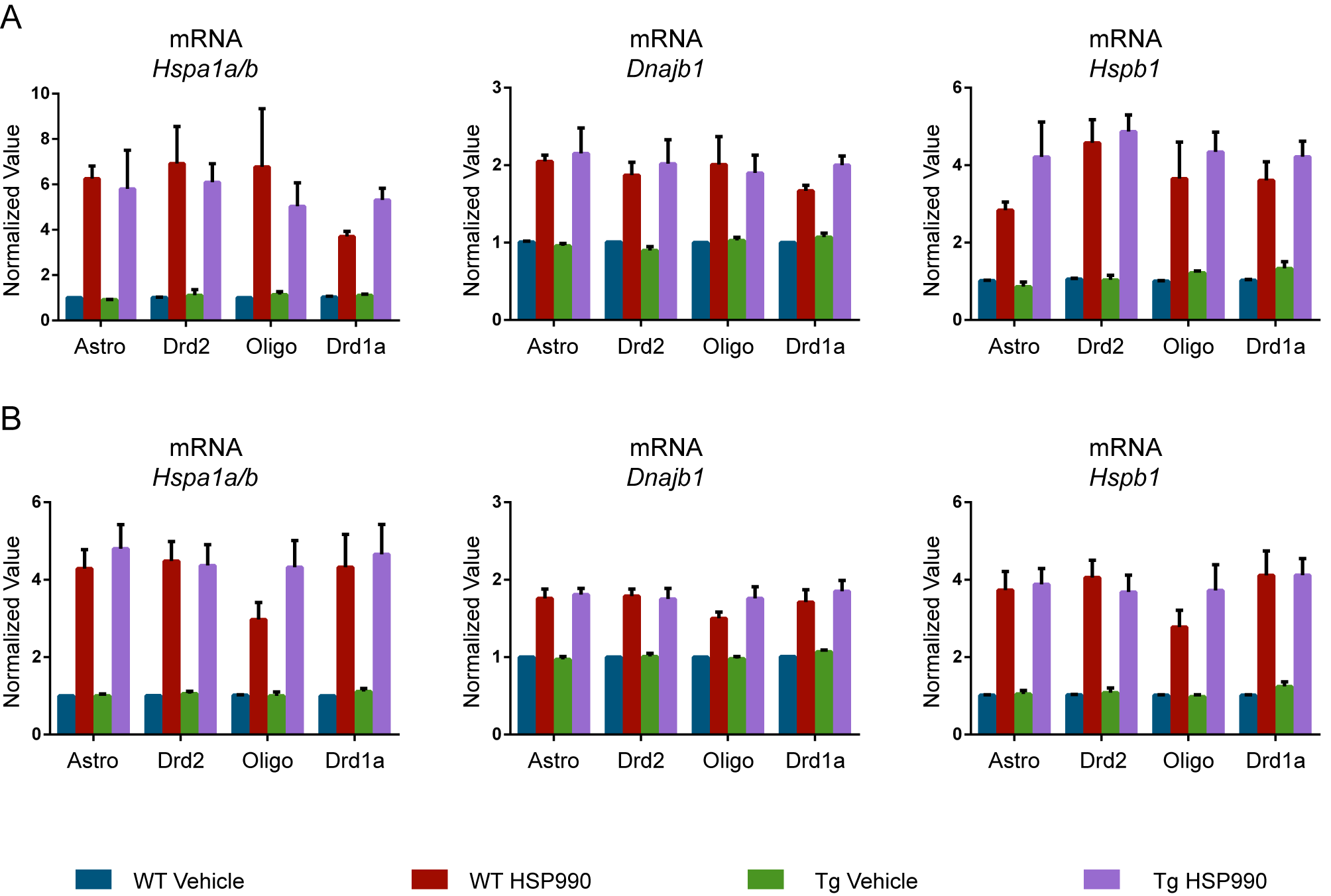

Supplement: S1 Fig — Hippocampal tissue was isolated from wild type and transgenic mice at 6 weeks of age 2 hours after treatment with HSP990 (12 mg/kg) or vehicle. RT-qPCR analysis of the expression levels of Hspa1a/b, Dnajb1 and Hspb1 in (A) tissue collected from mice used for the mRNA analysis (Fig 1) and (B) tissue collected from mice used for the protein analysis (Fig 3). Values were calculated relative to vehicle treated wild type mice. WT = wild type, Tg = transgenic. Error bars are SEM. (TIF) [file pone.0131985.s002.tif]

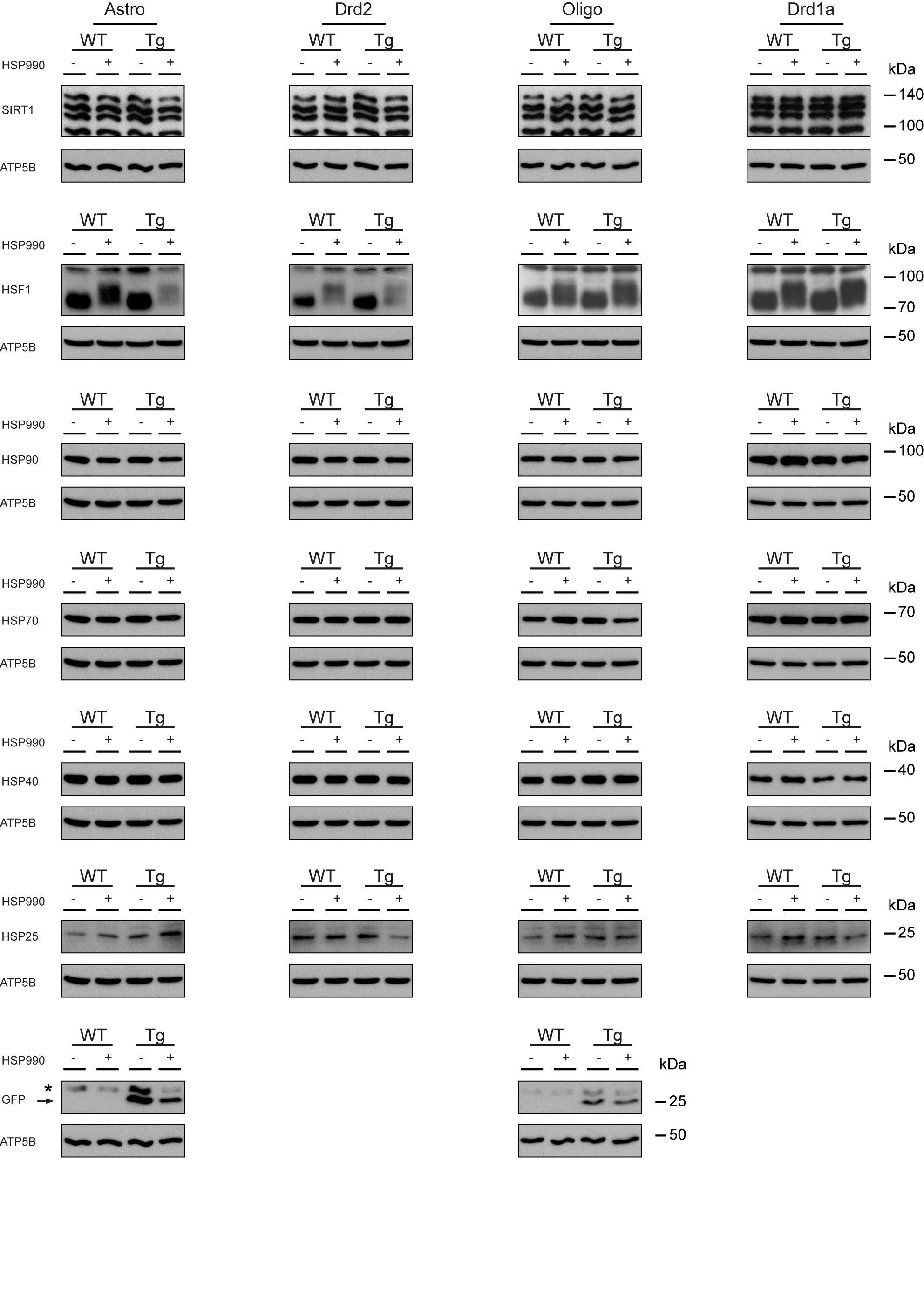

Supplement: S2 Fig — Hippocampal tissue was isolated from wild type and transgenic mice at 6 weeks of age 2 hours after treatment with HSP990 (12 mg/kg) or vehicle. Western blot analysis of the expression levels of SIRT1, HSF1, HSP90, HSP70, HSP40, HSP25 and GFP in mice treated with HSP990 as compared to those treated with vehicle. It was possible to use GFP to confirm the genotypes of the mice used for the Astro and Oligo lines, but GFP was not expressed in the hippocampus of the neuronal lines and so could not be used for this purpose. Loading control = ATP5B. WT = wild type, Tg = transgenic. *non-specific band. (TIF) [file pone.0131985.s003.tif]
