# Supplementary material for: In Vivo Profiling Reveals a Competent Heat Shock Response in Adult Neurons: Implications for Neurodegenerative Disorders
Source: PLoS One. 2015 Jul 2;10(7):e0131985. doi: 10.1371/journal.pone.0131985 (PMC4489736; doi:10.1371/journal.pone.0131985)
Supplement: S1 Table — (DOCX) [file pone.0131985.s004.docx]

**S1 Table**

**The total number of mice used to generate the number of GFP+ sorted cells that were pooled for the RNA and for the protein experiments.**

| **RNA analysis** | | | |
| --- | --- | --- | --- |
| **Mouse line** | **# exp** | **# mice** | **# GFP^+^ cells (total)** |
| Astro | 2 | 24 | Vehicle: 455,156  HSP990: 359,644 |
| Drd2 | 4 | 26 | Vehicle: 178,937  HSP990: 193,239 |
| Oligo | 3 | 36 | Vehicle: 303,093  HSP990: 255,079 |
| Drd1a | 4 | 52 | Vehicle: 232,418  HSP990: 221,551 |
| **Protein analysis** | | | |
| **Mouse line** | **# exp** | **# mice** | **# GFP^+^ cells (total)** |
| Astro | 7 | 84 | Vehicle: 1,309,086  HSP990: 1,318,902 |
| Drd2 | 7 | 94 | Vehicle: 407,937  HSP990: 423,154 |
| Oligo | 5 | 64 | Vehicle: 492,036  HSP990: 677,146 |
| Drd1a | 8 | 100 | Vehicle: 291,434  HSP990: 237,256 |
